# Atrial fibrillation prediction in patients with hypertrophic cardiomyopathy based on long-term follow-up data and machine learning model

**DOI:** 10.3389/fphys.2026.1814593

**Published:** 2026-06-08

**Authors:** Wan-xuan Ding, Guo-cao Li, Hao-yu Dong, Da-fei Zong, Xin-jing Ai, Yun-long Xia, Xiao-lei Yang, Ying-xue Dong

**Affiliations:** Department of Cardiology, The First Affiliated Hospital of Dalian Medical University, Dalian, China

**Keywords:** atrial fibrillation, hypertrophic cardiomyopathy, machine learning, prediction, P-wave indices

## Abstract

**Background:**

Hypertrophic cardiomyopathy (HCM) significantly increases the risk of new-onset atrial fibrillation (AF) through distinct pathogenic mechanisms. This study develops a machine learning (ML) model to improve AF prediction in patients with HCM and investigates electrophysiological abnormalities and non-traditional factors as preclinical predictors. The findings aim to inform early warning systems and precision management strategies for AF in this patient cohort.

**Methods:**

This retrospective cohort study consecutively enrolled patients diagnosed with HCM who had no prior history of AF from 2014 to 2023. A two-step feature selection procedure involving the Boruta algorithm and LASSO regression was implemented, and four machine-learning based algorithms were trained and compared against the reference model of the HCM-AF score. The Shapley Additive Explanations (SHAP) method was utilized for interpretability.

**Results:**

A total of 225 patients (annual incidence ratio 3.42%, 225/1014) developed new-onset AF during the median follow-up period of 6.50 years. Twelve clinical variables were identified through the feature selection process, with the random forest model demonstrating the best overall performance with area under the receiver-operating characteristic curve (AUC) 0.770 and 95% confidence interval (CI) of 0.699-0.840, outperforming the reference model (AUC 0.692, 95% CI 0.598-0.786) (Delong’s *P* = 0.012). The SHAP analysis revealed that indices of heart failure, age, left atrial size, followed by a history of cardiac implantable electronic devices, frequent atrial premature contractions, P-wave terminal force in lead V_1_, P-wave duration, PR interval, and left ventricular remodeling were associated with a significantly higher model-predicted risk of AF.

**Conclusions:**

The Random Forest model outperformed the HCM-AF score with robust predictive ability, validating P-wave indices as preclinical AF biomarkers through ML model for the first time. This integrated model offers a practical tool for early AF risk stratification in HCM, highlighting the clinical value of early symptomatic monitoring. Future multicenter validation is needed.

## Graphical Abstract

**Figure:**
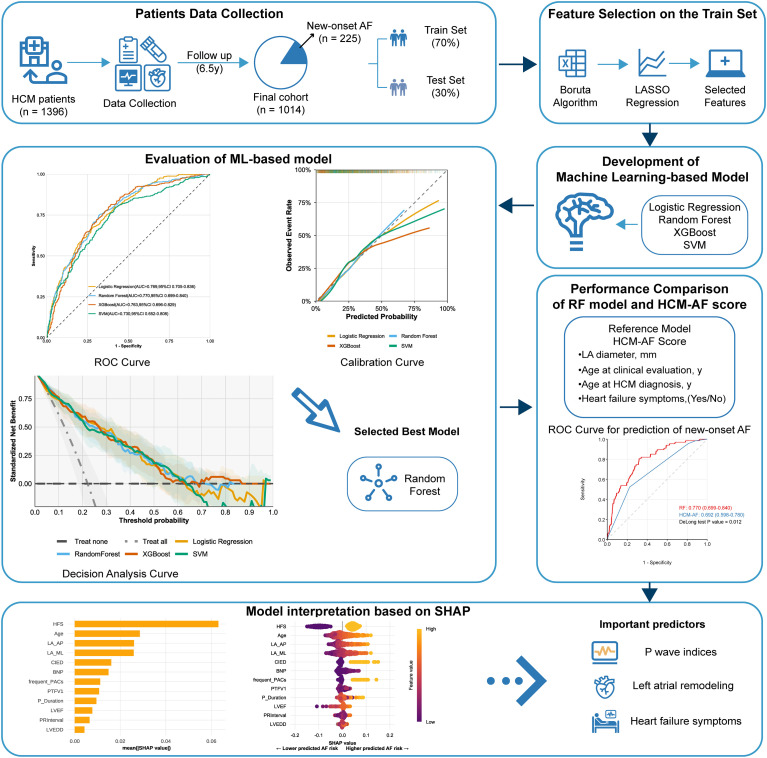
Development, validation, and performance comparison of machine learning models for predicting incident atrial fibrillation in patients with hypertrophic cardiomyopathy.

## 1 Introduction

Atrial fibrillation (AF) is highly prevalent in patients with hypertrophic cardiomyopathy (HCM), with its incidence rising to 40% in individuals aged 70 years and older (Siontis et al., 2014). AF serves as a pivotal outcome-modifying comorbidity in HCM patients, contributing to impaired cardiac function and elevated risks of thromboembolism and all-cause mortality. Several AF predictive models, including the CHARGE-AF and C_2_HEST scoring systems, had been developed for the general population and demonstrated favorable predictive performance (Alonso et al., 2013; Li et al., 2019). However, these models did not incorporate HCM-specific risk factors. As the earliest specific risk prediction model for AF in patients with HCM, the HCM-AF score showed improved predictive performance by integrating HCM-specific risk factors (Carrick et al., 2021); nevertheless, the limited sensitivity and specificity restricted widespread clinical implementation. Subsequent machine learning (ML) models achieved predictive performance gains, however the observed objects predominantly focused on cardiac structural and functional alterations, as well as hemodynamic variables, with less attention paid to atrial electrophysiological characteristics (Lu et al., 2025).

Atrial electrophysiological abnormalities, which can be effectively identified by non-invasive electrocardiographic assessments, are reliable and sensitive clinical surrogates for AF development (Chen et al., 2022). Cumulative evidence has confirmed that P-wave indices (PWIs) are robust predictors of AF onset, and our previous study further identified them as potential biomarkers for thromboembolism (Rasmussen et al., 2020; Dong et al., 2023). Notably, these indices are easily accessible and capable of detecting electrophysiological alterations before the occurrence of overt cardiac structural changes. Based on foundational research, the present study aimed to develop a clinically feasible AF risk prediction model for patients with HCM. We also sought to clarify whether electrophysiological abnormalities or other non-traditional factors, beyond age and left atrial (LA) size, the predominant predictors in the general population, can serve as preclinical biomarkers for AF in this high-risk cohort. The overall research process is shown in Graphical Abstract.

## 2 Methods

### 2.1 Study population and data collection

This retrospective cohort study consecutively enrolled adult patients with HCM admitted to the inpatient departments of our center from January 2014 to December 2023. Patients with alternative causes of myocardial hypertrophy—including hypertension, aortic stenosis, infiltrative cardiomyopathies, and athlete’s heart—were excluded based on clinical history and imaging findings, with laboratory or genetic evaluations conducted as clinically indicated. Furthermore, patients who had undergone septal reduction therapy or had a history of AF or atrial flutter were excluded from the study cohort. The study protocol received approval from the Institutional Ethics Committee of our hospital.

Clinical data were extracted from electronic medical records at enrollment, including demographic characteristics, family history of HCM, comorbidities, cardiac electrical parameters from 12-lead electrocardiograms (ECGs), medication regimens, laboratory results, and transthoracic echocardiographic (TTE) parameters. PWIs, including P-wave terminal force in lead V_1_ (PTFV_1_), P-wave duration (PWD), P-wave axis (PWA), and advanced interatrial block (A-IAB), were automatically measured using the GE Marquette™ 12SL™ ECG Analysis System (GE Marquette, Milwaukee, WI, USA). All automated measurements were independently validated by two experienced cardiologists, and manual remeasurements were subsequently performed by two independent ECG-specialized cardiologists to ensure data accuracy.

Echocardiographic analyses were completed at our hospital’s Echocardiography Reading Center in accordance with the recommendations of American Society of Echocardiography. Transthoracic echocardiography was performed in all participants using the Vivid 7 Ultrasound System (GE Vingmed Ultrasound, Horten, Norway). Left ventricular ejection fraction (LVEF), LA diameter, and other relevant parameters were assessed via TTE within 24 hours of admission. LA anteroposterior diameter was measured in the parasternal long-axis view, while mediolateral and superoinferior diameters were obtained from the apical four-chamber view. All TTE measurements and interpretations were conducted by experienced attending physicians.

### 2.2 Definition and criteria

The diagnosis of HCM was established in accordance with the 2024 American Heart Association/American College of Cardiology Guidelines for the Diagnosis and Management of HCM (Ommen et al., 2024). It was defined as an end-diastolic left ventricular wall thickness of ≥15 mm in adult patients without a family history of HCM, or ≥13 mm in those with a positive family history. Non-obstructive HCM was characterized by a peak left ventricular outflow tract (LVOT) gradient of <30 mmHg at rest and during physiological provocation.

Clinical AF was defined as the presence by typical symptoms (e.g., palpitations, dyspnea, fatigue, lightheadedness) or AF-associated clinical events (e.g., ischemic stroke), and Heart failure symptoms (HFS) were defined as the presence of at least one typical symptom, including dyspnea, orthopnea, paroxysmal nocturnal dyspnea, reduced exercise tolerance, fatigue, and prolonged recovery time after exercise (McDonagh et al., 2021; Ommen et al., 2024). Asymptomatic AF episodes detected by ambulatory ECG monitoring or cardiac implantable electric device (CIED) interrogation, lasting less than six minutes, were excluded from the final analysis (Doehner et al., 2025). The HCM-AF risk scores were calculated using a point system (Carrick et al., 2021).

PTFV_1_ was calculated as the product of the duration (in milliseconds) and the absolute value of the depth (in microvolts) of the negative terminal component of the median P-wave in lead V_1_. PWD was defined as the longest P-wave duration across all 12 ECG leads (Huang et al., 2020). P-wave axis (PWA) was directly extracted from the GE system, with abnormal PWA (aPWA) defined as a measurement <0° or >75°. P-wave dispersion was defined as the difference between the maximum and minimum P-wave durations measured across all 12 ECG leads (Chen et al., 2022).

### 2.3 Patients follow-up

The primary endpoint of this study was the new-onset clinical AF. Follow-up data were retrieved from the institutional electronic medical record system. Because of the retrospective design, AF detection was based on routine clinical follow-up rather than a standardized screening strategy. For patients without documented AF diagnoses during routine follow-up, telephone or outpatient follow-up was conducted to confirm whether AF had been diagnosed through 12-lead ECG, ambulatory ECG monitoring, or CIED interrogation at external medical institutions, as well as to verify vital status. The follow-up period for each patient commenced on the date of enrollment and concluded at the earliest occurrence of the following events: diagnosis of new-onset AF, all-cause death, or the administrative censoring date of December 31, 2025.

### 2.4 Development of machine learning model and performance evaluation

All patients were randomly allocated to the training and test set in a 7:3 ratio. To ensure data quality, variables with more than 20% missing values were excluded before imputation. Before imputation, categorical and continuous predictors were coded as factor and numeric variables, respectively. Missing data in the remaining variables were imputed according to their original variable types using the missRanger package in R software, with imputation performed separately for the training and test sets to avoid information leakage, resulting in five imputed training sets and five imputed test sets. Model construction and feature selection were restricted to the imputed training sets to prevent overfitting.

Feature selection was conducted in two stages. First, the Boruta algorithm was utilized to identify all clinically and statistically relevant features. Subsequently, variables deemed important by the Boruta algorithm were further refined using the least absolute shrinkage and selection operator (LASSO) regression. Variables retained by LASSO regression in the majority of imputations (≥3/5) were defined as consensus features, which were then used for hyperparameter tuning and ML model development.

Four ML models were implemented: Logistic Regression (LR), Random Forest (RF) and Extreme Gradient Boosting (XGBoost), and Support Vector Machine (SVM). Hyperparameters were tuned via repeated 10-fold cross-validation (five repetitions) within the training sets. Optimal classification thresholds for ML models were determined in the training set using the Youden index during cross-validation and then applied to the test set to calculate threshold-dependent performance metrics, including sensitivity, specificity, accuracy, positive predictive value (PPV), and negative predictive value (NPV).

Model performance was assessed separately in each imputed test set, and metrics were pooled using Rubin’s rules. Discriminative ability was evaluated via the area under the receiver operating characteristic curve (AUC), along with sensitivity, specificity, and accuracy. Calibration was measured using the Brier score and visualized with calibration plots, and clinical utility was assessed using decision curve analysis (DCA). The final optimal model was selected based on a comprehensive assessment of discriminative ability, calibration accuracy, and clinical net benefit. The HCM-AF score, incorporating four clinical variables (LA dimension, heart failure symptoms, age at HCM diagnosis, and age at clinical evaluation), was established as the reference model. The performance of the ML model and the HCM-AF score was compared using AUC, sensitivity, specificity, accuracy, PPV, and NPV. AUC differences were assessed using DeLong’s test.

To enhance the interpretability of the final selected model, the Shapley Additive exPlanations (SHAP) framework was applied across the imputed test sets to quantify the global contribution of each feature on the model’s predictions.

### 2.5 Statistical analysis

All analyses were performed using R software (version 4.3.3). Continuous variables were assessed for normality using the Shapiro-Wilk test. Normally distributed variables were expressed as mean ± standard deviation and compared using the independent two-sample t-test. Non-normally distributed variables were presented as median (interquartile range [IQR]) and analyzed using the Mann-Whitney U test. Categorical variables were expressed as frequencies (percentages) and compared using the chi-square test or Fisher’s exact test, as appropriate. *P* value < 0.05 was considered statistically significant.

## 3 Results

### 3.1 Baseline characteristics

**Figure 1:**
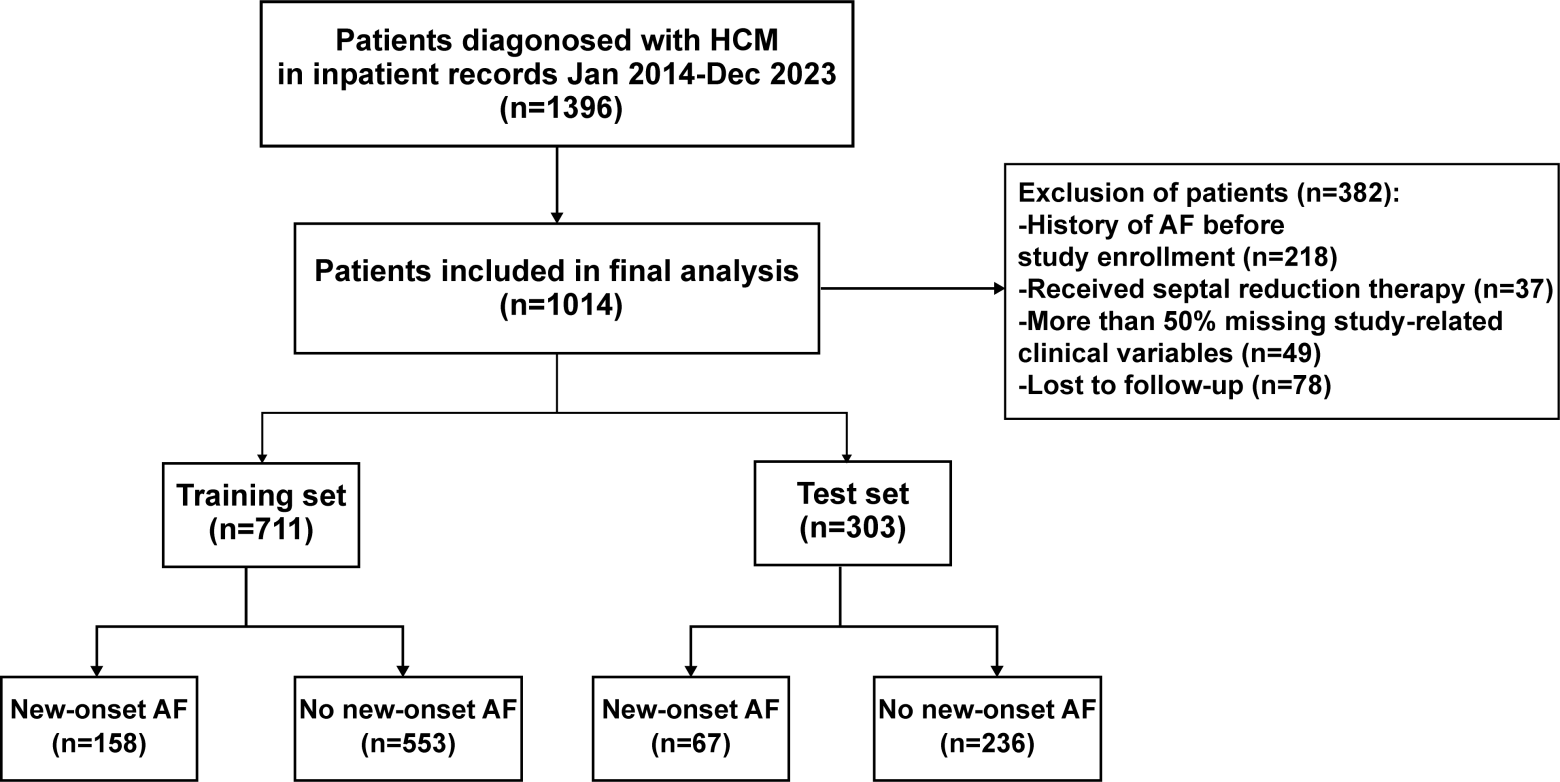
Flowchart of patient inclusion and allocation. AF, atrial fibrillation; HCM, hypertrophic cardiomyopathy.

A total of 1,396 patients with HCM were initially enrolled in this study. After excluding 218 patients with a prior diagnosis of AF, 37 who had undergone septal reduction therapy before enrollment or during follow-up, 49 with more than 50% missing baseline data relevant to the study, and 78 lost to follow-up, 1,014 patients were finally included in the definitive analysis. The median follow-up duration for the study cohort was 6.50 years (IQR: 4.11–9.40 years). The patient selection process is illustrated in **Figure 1**. **Supplementary Figure 1** shows the missing percentages of candidate variables before imputation. Variables with more than 20% missing values were excluded before model development, including prior maximum systolic blood pressure, prior maximum diastolic blood pressure, left ventricular outflow tract peak systolic velocity, and N-terminal pro-B-type natriuretic peptide (NT-proBNP).

Of the included patients, 225 (22.19%) developed new-onset AF during the follow-up period. The class distribution in the training set and test set is shown in **Supplementary Figure 2**. Baseline clinical characteristics of the cohort stratified by the occurrence of new-onset AF are summarized in **Supplementary Table 1**. Significant intergroup differences were observed in multiple variables, including age, family history of HCM, HF status, B-type natriuretic peptide (BNP) levels, and the frequency of premature atrial contractions (PACs) and premature ventricular contractions (PVCs). Additionally, notable variations were detected in cardiac structural and functional parameters such as LVEF, E/e’ ratio, and left atrial diameter. Importantly, electrocardiographic markers—particularly QRS duration and P-wave indices including PTFV_1_, P-wave dispersion, A-IAB, and PWD—also exhibited significant between-group differences.

### 3.2 Feature selection via Boruta algorithm and LASSO regression

**Figure 2:**
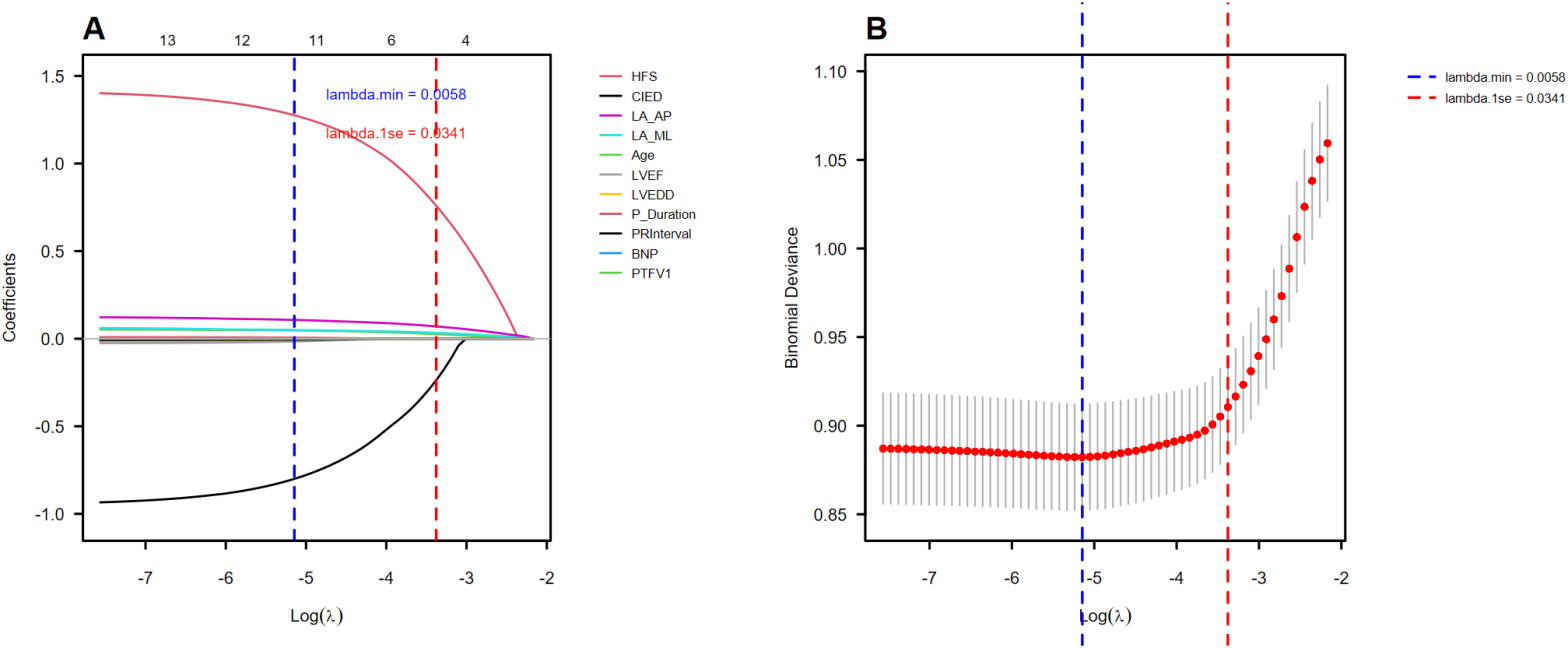
Feature selection via LASSO regression. **(A)** LASSO coefficient path plot: Coefficient paths of candidate predictors across log-transformed penalty parameters (log λ). **(B)** Cross-validation curve for LASSO regression: the blue and red dashed lines denote the λmin (0.0058) and λ1se (0.0341) respectively.

In the training cohort (n=711), a two-step feature selection approach was adopted. First, the Boruta algorithm was independently applied to each imputed training dataset to identify potential predictors of AF from baseline variables. Subsequently, both confirmed and tentative features identified by Boruta were incorporated into a LASSO regression model for further predictor refinement. To ensure feature stability across imputations, variables retained by LASSO in at least 60% of the five imputed training datasets were defined as consensus features, yielding a stable set of 12 features for model development. These features included age, BNP levels, presence of HF symptoms, frequent PACs, left atrial anteroposterior diameter (LA-AP), left atrial mediolateral diameter (LA-ML), CIED implantation, LVEF, left ventricular end-diastolic diameter (LVEDD), PR interval, PWD, and PTFV_1_. **Supplementary Figure 3** illustrates the aggregated feature importance derived from the Boruta algorithm across all imputations, and **Figure 2** depicts the LASSO coefficient shrinkage paths and cross-validation curve, with the first imputed training set (D1-train) as a representative example. Full terms of feature labels and abbreviations used in the figures are provided in **Supplementary Table 2**.

### 3.3 Construction and performance evaluation of machine learning models

**Figure 3:**
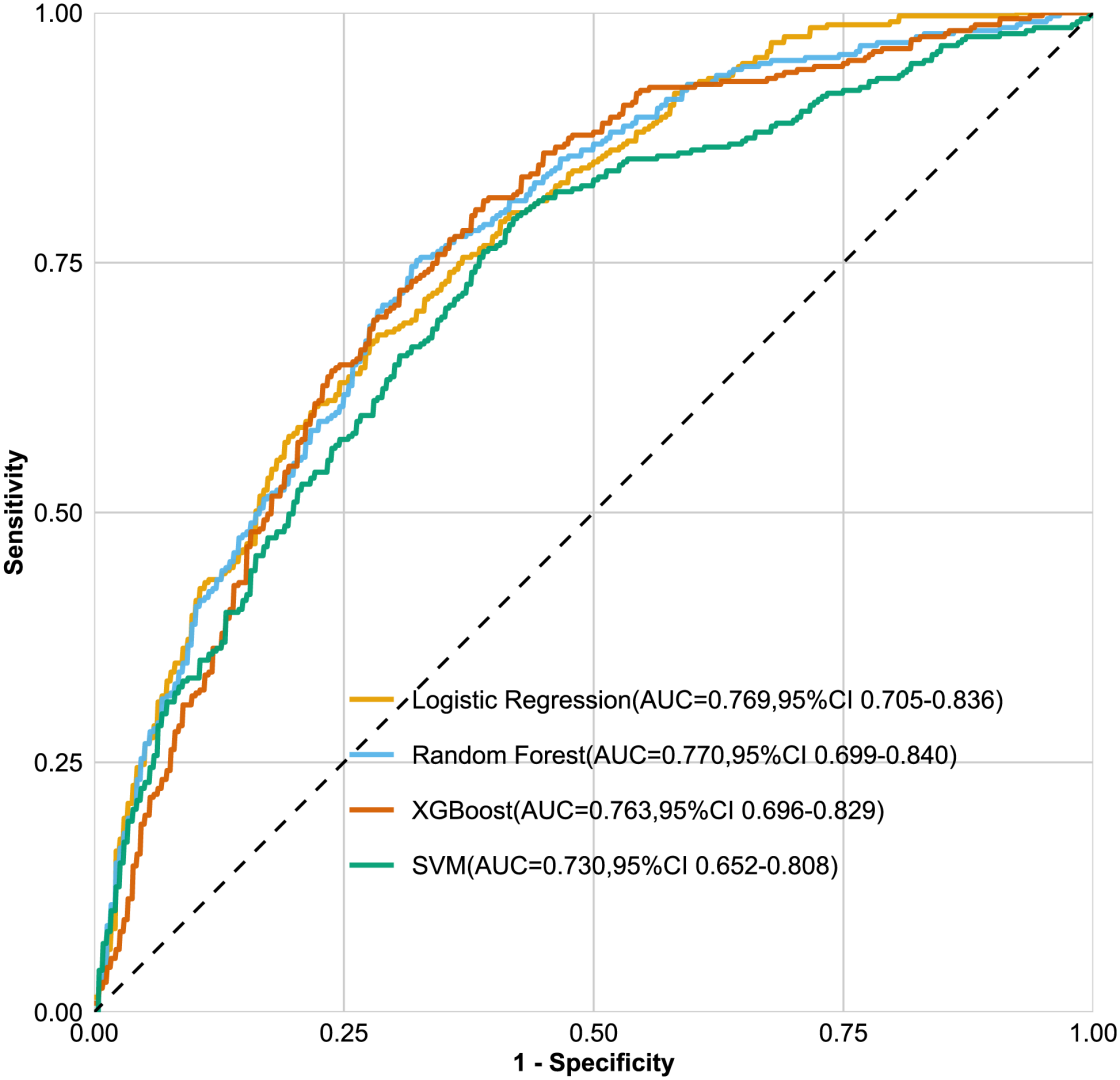
ROC curves of four machine learning models based on the test set. SVM, support vector machine.

Based on the consensus features identified during feature selection, four machine learning models (LR, RF, XGBoost, and SVM) were developed and optimized. The discriminative ability of the models was assessed using receiver operating characteristic (ROC) curve analysis based on the test set (**Figure 3**), with ROC curves based on the training set provided in **Supplementary Figure 4**. All models demonstrated reliable discriminatory performance. Among these, the RF model achieved the highest AUC of 0.770 (95% CI: 0.699–0.840). Detailed performance metrics for each model across the multiple imputed datasets are presented in **Table 1**.

**Table 1:** Performance of four machine learning models for predicting incident AF in HCM patients.

Models	AUC (95%CI)	Sensitivity	Specificity	Accuracy	Brier score	NPV	PPV
Logistic Regression	0.769 (0.705–0.836)	0.702 (0.570–0.833)	0.679 (0.614–0.744)	0.684 (0.627–0.740)	0.146 (0.121–0.170)	0.889 (0.838–0.941)	0.383 (0.293–0.472)
Random Forest	0.770 (0.699–0.840)	0.716 (0.600–0.833)	0.694 (0.590–0.798)	0.699 (0.619–0.779)	0.145 (0.122–0.169)	0.896 (0.851–0.942)	0.401 (0.299–0.504)
XGBoost	0.763 (0.696–0.829)	0.709 (0.616–0.838)	0.664 (0.548–0.779)	0.682 (0.602–0.763)	0.151 (0.124–0.177)	0.904 (0.855–0.952)	0.390 (0.292–0.487)
Support Vector Machine	0.730 (0.652–0.808)	0.684 (0.544–0.823)	0.658 (0.578–0.737)	0.663 (0.601–0.725)	0.154 (0.127–0.181)	0.880 (0.827–0.934)	0.362 (0.273–0.451)

AF, atrial fibrillation; HCM, hypertrophic cardiomyopathy; AUC, area under the curve; CI, confidence interval; NPV, negative predictive value; PPV, positive predictive value; SVM, support vector machine.

**Figure 4:**
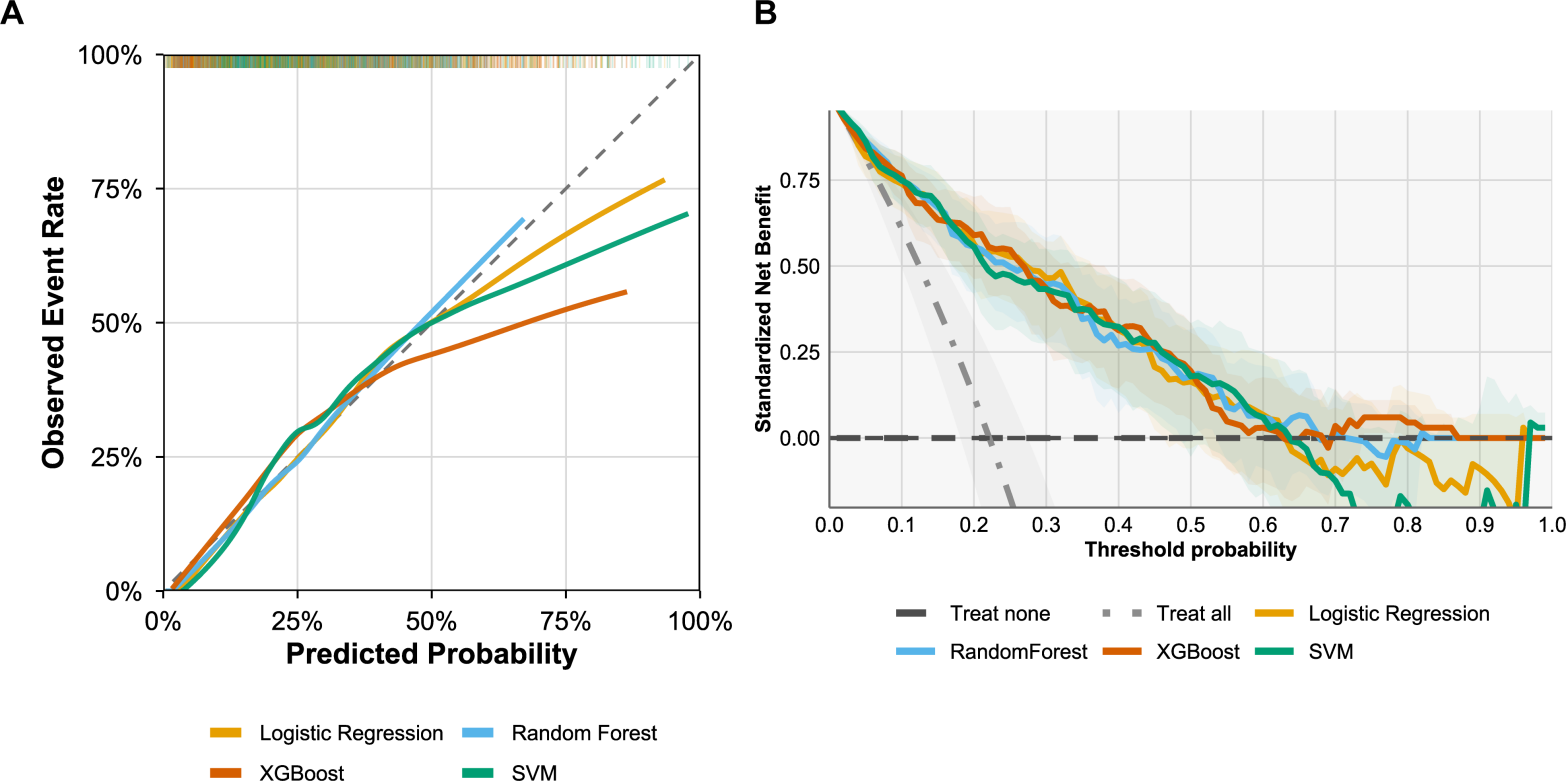
Calibration and clinical utility evaluation of machine learning models. **(A)** Calibration curves for four machine learning models: Logistic Regression, Random Forest, XGBoost, and SVM. **(B)** Decision curve analysis for the four ML models. Net benefit is plotted across threshold probabilities; the gray reference lines represent the “treat-none” and “treat-all” strategies. SVM, support vector machine; ML, machine learning.

All models exhibited favorable calibration, with predicted probabilities showing good concordance with observed event rates; notably, the RF model demonstrated the closest alignment with the ideal calibration line (**Figure 4A**). DCA revealed that all models provided a positive net benefit across a range of threshold probabilities when compared with the treat-all and treat-none strategies (**Figure 4B**). In particular, the RF model consistently yielded the highest net benefit across most threshold probability ranges. In the independent test cohort (n=303), the RF model exhibited the most favorable and balanced predictive performance, characterized by the highest AUC, optimal calibration, consistently high net benefit across a broad range of threshold probabilities, and well-balanced sensitivity and specificity (**Table 1**). Consequently, the RF model was selected as the final model for further interpretive analysis. The optimal classification threshold for the final RF model was 0.318, as determined by the Youden index in the training set.

### 3.4 Performance comparison of the Random Forest model and the HCM-AF score

ROC curves for AF prediction in the test sets generated by the RF model and the HCM-AF score are shown in **Supplementary Figure 5**. After pooling performance across the five imputed test sets using Rubin’s rules, the AUC of the RF model was 0.770 (95% CI: 0.699–0.840), compared with 0.692 (95% CI: 0.598–0.786) for the HCM-AF score. DeLong’s test confirmed a statistically significant difference in AUC between the two models (*P* = 0.012). Additionally, the RF model outperformed the HCM-AF score across all key performance metrics, including sensitivity, specificity, accuracy, PPV, and NPV (**Table 2**).

**Table 2:** Test-set performance comparison between the Random Forest model and the HCM-AF score for AF prediction in patients with HCM.

Prediction model	AUC (95%CI)	Sensitivity	Specificity	Accuracy	NPV	PPV	Delong *P*-value
Random Forest model	0.770 (0.699–0.840)	0.716 (0.600–0.833)	0.694 (0.590–0.798)	0.699 (0.619–0.779)	0.896 (0.851–0.942)	0.401 (0.299–0.504)	0.012
HCM-AF score	0.692 (0.598–0.786)	0.696 (0.567–0.824)	0.676 (0.549–0.804)	0.680 (0.585–0.776)	0.887 (0.837–0.936)	0.382 (0.271–0.494)	

AF, atrial fibrillation; HCM, hypertrophic cardiomyopathy; AUC, area under the curve; CI, confidence interval; NPV, negative predictive value; PPV, positive predictive value.

### 3.5 Model interpretation via SHAP analysis

**Figure 5:**
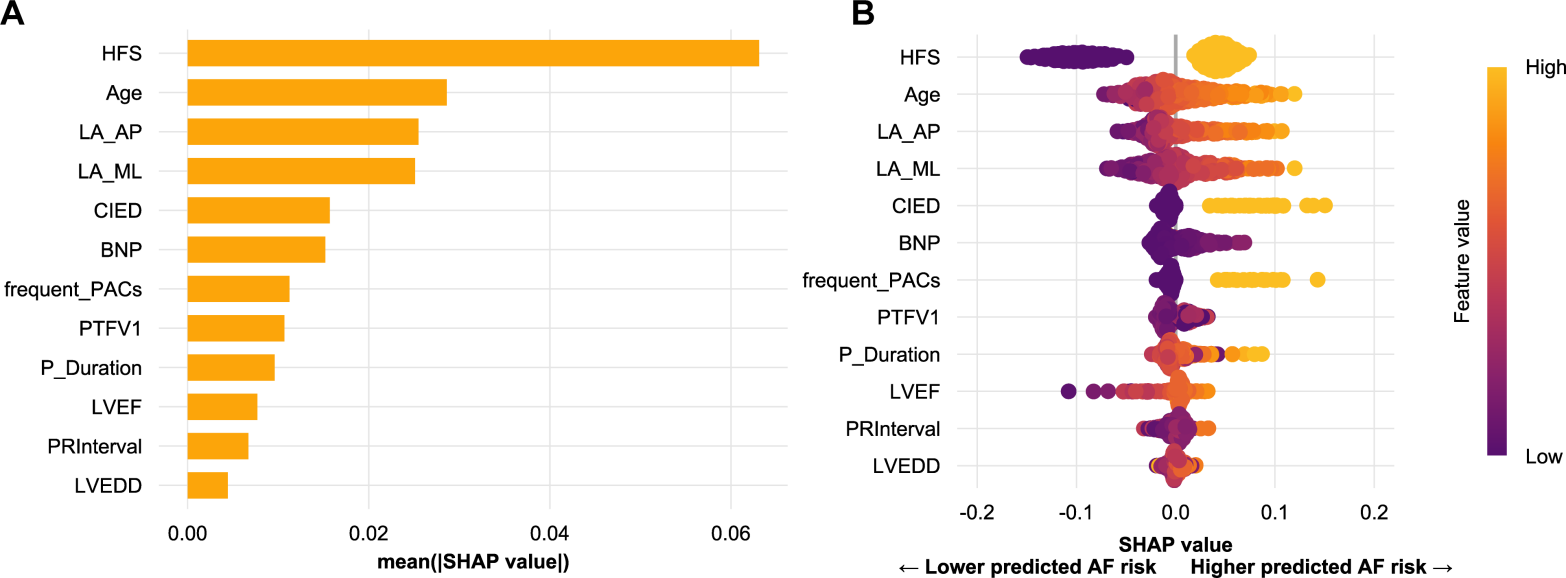
SHAP-based interpretation of the Random Forest model in the D1 test set. **(A)** Mean absolute SHAP value ranking of feature importance. **(B)** SHAP beeswarm plot showing the contribution and direction of each feature to individual predictions.

To enhance the interpretability of the top-performing RF model, SHAP analysis was conducted across the five imputed test sets (D1–D5), and **Figure 5** shows the results from the D1 test set as a representative example. The corresponding SHAP plots for the D1 training set are provided in **Supplementary Figure 6**. Across these imputed test sets, the overall ranking of feature importance exhibited high consistency, with only minor variations observed in the rankings of moderately important features, such as BNP levels and frequent PACs. SHAP analysis demonstrated that HF symptoms, age, LA size, electrocardiographic indices (including PTFV_1_, PWD, frequent PACs and PR interval), along with LVEF, BNP level, and left ventricular (LV) size were collectively associated with an increased model-predicted risk of AF.

## 4 Discussion

### 4.1 Main findings

Approximately 22.19% of patients with HCM developed new-onset AF during a median follow-up period of 6.50 years. Compared to the reference model for HCM-AF and other predictive models, the RF model demonstrated superior performance in predicting new-onset AF in patients with HCM. The risks of AF varied significantly, with HF symptoms playing a crucial role alongside the well-established predictors of age and LA size. Furthermore, this study validated for the first time that electrical abnormalities serve as preclinical indicators of AF through ML model in this high-risk patient cohort.

### 4.2 Clinical importance of predicting AF in HCM

AF incidence is markedly elevated in HCM patients relative to the general population, with an annual incidence of approximately 5% (Choi et al., 2018); consistent with this, our study found a cumulative new-onset AF incidence of 22.19% over 6.50 years of follow-up. Beyond increased AF risk, HCM confers an approximately fourfold higher risk of thromboembolism in AF patients, even after adjustment for the CHA_2_DS_2_-VASc score (Hokuriku-Plus et al., 2019). Additionally, HCM patients experience significantly higher AF recurrence rates after catheter ablation (13.3%–92.9%) compared with non-HCM patients (7.6%–58.8%) across 13–54 months of median follow-up in existing studies (Ezzeddine et al., 2023).

Current clinical guidelines recommend prompt anticoagulation initiation in HCM patients with AF irrespective of CHA_2_DS_2_-VASc scores, and effective AF risk stratification in HCM is imperative given the heightened risk of HF and thromboembolism in those with comorbid HCM and AF. These unique clinical characteristics indicate that AF predictive factors in HCM differ from those in the general population, and traditional risk assessment approaches are insufficient for this cohort. By developing and validating ML models for HCM patients, this study addresses a critical gap in the clinical management of this population, providing a novel tool for targeted AF risk stratification and intervention.

### 4.3 Prediction performance of left cardiac electrical remodeling

AF and HCM share well-established overlapping pathogenic mechanisms, including myocardial structural remodeling, interstitial fibrosis, and neurohormonal activation. In HCM, LV diastolic dysfunction elevates LV filling pressures, which in turn increase LA pressure and drive LA structural remodeling (Zegkos et al., 2017). Notably, electrophysiological abnormalities, including conduction delays and electrical heterogeneity, typically manifest prior to overt structural changes; these derangements induce atrial electromechanical dyssynchrony, ultimately augmenting susceptibility to AF initiation and maintenance (Heijman et al., 2021).

ECG is a noninvasive, cost-effective, and widely accessible tool for detecting cardiac electrical anomalies, with PWIs serving as reliable surrogates for atrial electrical remodeling. A growing body of evidence links PWD and PTFV_1_ to incident AF (Huang et al., 2020; Zagoridis et al., 2023), and this study is the first to integrate these ECG-derived parameters into an ML framework to predict new-onset AF in HCM patients. Our analysis revealed a positive correlation between AF risk and PWD, PTFV_1_, and PR interval; while other PWIs (A-IAB and P-wave dispersion) were not included in the final model, statistically significant differences were observed for these metrics, supporting their potential clinical utility for HCM-related AF risk stratification.

Notably, AF predictive factors in HCM diverge from those in the general population, where advanced age and LA enlargement are dominant predictors (Raniga et al., 2024). We are the first to empirically test the hypothesis that electrical abnormalities constitute preclinical AF biomarkers in HCM via ML modeling, with SHAP analysis confirming an association between PWIs and new-onset AF in this cohort. Although PWI predictive performance was moderately inferior to LA dilation, this finding highlights a practical clinical trade-off: readily obtainable, low-cost ECG markers offer a valuable alternative to more expensive imaging-based biomarkers for AF risk assessment, particularly in settings with limited access to advanced imaging.

### 4.4 Prediction performance of left cardiac structure remodeling

Emerging evidence suggests HCM patients may present with primary atrial myopathy and fibrosis, independent of secondary hemodynamic mechanisms, an important pathological basis for AF susceptibility (Efremidis et al., 2021). LA enlargement is a well-recognized strong predictor of AF, as LA enlargement and fibrosis together create disorganized electrical conduction pathways, exacerbate atrial electrical remodeling, and further elevate AF risk (Ariyaratnam et al., 2024). Prior studies have identified an enlarged LA-AP as an independent risk factor for new-onset AF in HCM (Debonnaire et al., 2017; Kubo et al., 2021), but anatomical constraints from the sternum and spine limit anteroposterior LA expansion, leading to asymmetric LA enlargement that predominates in the superoinferior and mediolateral directions (Ballo et al., 2016).

To address the limitations of single-dimension LA measurement, this study integrated multiplanar linear LA dimensions for a more comprehensive assessment of LA structural remodeling. SHAP analysis revealed that LA-ML had comparable predictive importance to LA-AP, indicating that the directional pattern of LA enlargement carries clinical significance for AF risk stratification. This finding can refine imaging-based risk assessment and support the development of earlier preventive strategies for HCM patients. While current imaging guidelines advocate for LA volume/volume index (LAV/LAVI) as the gold standard for LA size assessment, multiplanar linear measurements represent a valuable and accessible alternative in clinical settings where volumetric data are unavailable (Lang et al., 2015).

Regarding ventricular geometry, previous research by Tian et al. identified LV remodeling indices as independent predictors of AF. LVEDD was similarly selected in our model (Tian et al., 2018). However, SHAP analysis showed LVEDD’s predictive contribution was relatively lower than atrial indices, a result consistent with HCM’s core pathophysiology, which is characterized by myocardial hypertrophy with normal or even reduced LV cavity size, while overt LV enlargement is a hallmark of end-stage disease (Marian and Braunwald, 2017; Rowin et al., 2020). Thus, while LVEDD contributes to AF risk stratification, its sensitivity for predicting early new-onset AF in HCM is limited.

### 4.5 Prediction performance of clinical symptom and left cardiac function

HCM is defined by distinct hemodynamic abnormalities: asymmetric LV hypertrophy, left ventricular outflow tract (LVOT) obstruction, and LV diastolic dysfunction (Marian and Braunwald, 2017). These derangements directly elevate LA filling pressures and cause sustained atrial pressure overload, a key upstream trigger of atrial electrical and structural remodeling. The LVOT gradient is a critical hemodynamic marker in HCM, closely linked to clinical symptoms, HF, and adverse prognosis, but its association with AF remains controversial in existing literature (Maron et al., 2003; Ommen et al., 2024). Small-sample studies have suggested a potential correlation (Cergan et al., 2025), but two large-scale multicenter cohort studies (4,248 and 2,755 patients, respectively) consistently demonstrated the LVOT gradient is not an independent AF predictor after adjustment for LA diameter and other clinical covariates (Guttmann et al., 2017; Kramer et al., 2021). Our analysis aligned with these findings, showing no significant association between LVOT obstruction-related hemodynamic parameters (LVOT peak velocity, systolic anterior motion [SAM] of the mitral valve) and new-onset AF.

This lack of independent association is explained by HCM’s AF pathophysiological cascade: the LVOT gradient acts as an early upstream driver that promotes AF indirectly by exacerbating LA remodeling (via increased LV filling pressures and mitral regurgitation), rather than directly triggering AF (Williams et al., 2015). Before the gradient induces significant atrial electrical/structural changes, it is insufficient to initiate AF; additionally, LA size, as a downstream marker of atrial remodeling, captures the cumulative effect of LVOT obstruction on the atria, making it a far stronger predictor. For this reason, the LVOT gradient’s predictive value is attenuated or masked when LA size is included in multivariable models.

LVEF is a universal index of global LV systolic function, and retrospective studies link reduced LVEF to AF in HCM (Sivalokanathan et al., 2019), likely because declining LVEF signals more severe myocardial disease, which secondarily elevates AF risk. Consistent with this, our analysis found significantly lower LVEF in the new-onset AF group vs. the non-AF group, and LVEF was included in the final model. However, SHAP analysis showed its feature importance was relatively low, as LVEF is preserved or mildly elevated in most HCM patients, with significant declines occurring only in advanced disease stages—after AF onset in most cases (Olivotto et al., 2012). Thus, LVEF has lower sensitivity for early AF risk stratification compared with LA markers that reflect early atrial remodeling.

Notably, SHAP analysis identified HF symptoms as a powerful predictor of new-onset AF in our model, a finding with key clinical implications. In HCM, AF typically develops prior to overt HF (as LVEF remains preserved until late-stage disease); further, AF and HF share overlapping symptoms (Al-Khatib et al., 2020), and AF detection/diagnosis often lags behind symptom onset. Collectively, these factors mean HF symptoms can serve as an early warning signal for subclinical or imminent new-onset AF in HCM. This demonstrates that effective AF risk stratification does not rely solely on imaging features (e.g., myocardial scarring, LA enlargement); capturing early symptomatic changes is equally valuable, rendering our model a cost-effective, widely accessible tool for routine clinical practice.

Pharmacological therapy is the cornerstone of HCM hemodynamic management, and beyond traditional agents, cardiac myosin inhibitors (CMIs) represent a novel avenue for improving cardiac structural remodeling. Emerging research shows aficamten significantly reduces LA volume without increasing AF risk (Rowin et al., 2026), likely by improving LV diastolic compliance and alleviating LA pressure overload—directly targeting the pathophysiological pathway of HCM-related AF. While this finding is promising, further long-term clinical trials are warranted to validate the causal relationship and clinical benefit of CMIs for AF prevention in HCM.

### 4.6 ML model development and clinical translation

Most existing AF risk prediction models for HCM rely on traditional linear regression methods, which are inherently limited for this population (Carrick et al., 2021; Lu et al., 2025): AF risk in HCM is driven by a complex interplay of clinical, electrophysiological, and imaging factors with nonlinear associations and interactive effects, which linear regression cannot fully capture. This critical limitation highlights the necessity of ML approaches, which construct predictive models without *a priori* assumptions about variable relationships, making them far better suited to model HCM’s complex AF pathophysiology.

Among the four ML models developed in this study, the RF model exhibited superior performance across all evaluation metrics, with reliable discriminative ability, robust calibration, and significant clinical net benefit. Compared with the HCM-AF score (AUC 0.692), the RF model achieved a higher AUC of 0.770, alongside improved sensitivity, specificity, and negative predictive value, suggesting improved predictive performance. This improvement may be related to the incorporation of early electrophysiological markers not captured by the HCM-AF score, together with the ability of the RF algorithm to model nonlinear relationships among predictors. The HCM-AF score mainly covers demographic characteristics, clinical symptoms, and left atrial structural remodeling, whereas the RF model additionally includes surface ECG markers related to atrial electrical remodeling and conduction delay, as well as frequent PACs reflecting atrial arrhythmic burden. These variables may provide earlier electrophysiological information before overt structural remodeling becomes evident and help refine individualized AF risk stratification. Beyond replicating known AF predictors, SHAP analysis identified PWIs as an important novel predictor in the model—a first in HCM-AF risk prediction research. This is clinically impactful because PWIs are derived from ECG, which offers distinct advantages over advanced imaging (speckle tracking echocardiography, cardiovascular magnetic resonance [CMR] feature tracking): noninvasiveness, low cost, high patient compliance, and convenient acquisition of cardiac electrophysiological information (e.g., atrial electrical remodeling). ECG is thus particularly suitable for large-scale AF screening and routine follow-up in HCM, addressing a key barrier to early AF detection.

By integrating ECG-derived PWIs with clinical and imaging features, our ML model demonstrates robust clinical utility for HCM-related AF prediction. It functions as both an effective screening tool to identify high-risk patients early and a decision-support tool to guide clinicians in formulating personalized monitoring and preventive strategies (e.g., targeted Holter monitoring, early anticoagulation assessment). This aligns with current clinical guidelines, which recommend periodic ECG and 24 to 48 hours Holter monitoring for HCM patients but leave the optimal frequency and duration of monitoring undefined (Ommen et al., 2024). Recent evidence shows extending monitoring duration or increasing frequency improves AF detection rates in high-risk HCM populations (Choe et al., 2015; Jawad-Ul-Qamar et al., 2020), and our model can complement this by identifying which patients would benefit most from enhanced monitoring—enabling targeted, cost-effective early intervention and timely anticoagulation therapy when indicated.

## 5 Limitations

This study has several important limitations that must be acknowledged. First, this is a single-center study based on hospitalized patients with HCM; therefore, selection bias cannot be fully excluded, and the generalizability of our findings to stable outpatient or community HCM populations should be interpreted with caution. In addition, AF detection was based on routine clinical follow-up rather than a standardized screening strategy, which may have led to underdetection of asymptomatic or paroxysmal AF. Second, the lack of external validation remains an important limitation. Validation in independent multicenter and geographically diverse cohorts is needed to confirm the robustness and generalizability of the RF model. Third, the developed model is a binary classification model that predicts cumulative AF risk over the entire follow-up period, rather than a time-to-event model; additionally, several potential high-value predictors (genetic factors, LAV/LAVI, late gadolinium enhancement [LGE] on CMR) were not included owing to their limited availability in this long-term retrospective cohort. While the model is valuable for AF risk stratification, it cannot estimate the precise timing of AF onset—a critical clinical gap that future research should address using time-dependent ML methods and integrating additional predictive variables.

## 6 Conclusions

This predicting model addresses critical gaps in traditional HCM management by capturing the complex nonlinear pathophysiology of HCM-related AF, enabling personalized patient monitoring and targeted preventive strategies. It also highlights the clinical value of early symptomatic monitoring (e.g., HF symptoms) and low-cost ECG markers for AF risk assessment, complementing traditional imaging-based approaches. Additionally, future large-scale, multicenter prospective studies integrating genetic factors, advanced imaging markers, and time-dependent ML methods are essential to improve its ability to predict the precise timing of AF onset, ultimately optimizing AF prevention and management in HCM patients.

## Data Availability

The original contributions presented in the study are included in the article/**Supplementary Material**. Further inquiries can be directed to the corresponding authors.
