# Supplementary material for: Atrial fibrillation prediction in patients with hypertrophic cardiomyopathy based on long-term follow-up data and machine learning model
Source: Front Physiol. 2026 Jun 8;17:1814593. doi: 10.3389/fphys.2026.1814593 (PMC13283817; doi:10.3389/fphys.2026.1814593)
Supplement: Supplementary file 1 [file DataSheet1.pdf]

Supplementary Material

1 Supplementary Tables

Supplementary Table 1. Baseline Characteristics of Patients.

	No new-onset AF (n=789)	new-onset AF (n=225)	*P* value
Age at clinical evaluation, years	60.03 ± 12.18	65.84 ± 11.77	<0.001
Age at HCM diagnosis, years	58.34 ± 12.92	62.98 ± 13.01	<0.001
Male, n (%)	506(64.13)	133(59.11)	0.169
Height, cm	168.56 ± 8.94	168.04 ± 8.45	0.437
Weight, kg	73.73 ± 12.44	72.59 ± 12.35	0.224
Smoking, n (%)	263 (33.33)	64 (28.44)	0.166
Drinking, n (%)	163 (20.66)	37 (16.44)	0.161
Hypertension, n (%)	407 (51.58)	126 (56.00)	0.242
Diabetes, n (%)	147 (18.63)	33 (14.67)	0.170
Coronary artery disease, n (%)	192 (24.33)	41(18.22)	0.055
Cerebral Hemorrhage, n (%)	8 (1.01)	3 (1.33)	0.966
Cerebral Infarction, n (%)	41 (5.20)	9 (4.00)	0.465
Hyperthyroidism, n (%)	8 (1.01)	2 (0.89)	1.000
Family history of HCM, n (%)	27 (3.42)	22 (9.78)	<0.001
Apical HCM, n (%)	123 (15.59)	21 (9.33)	0.018
Obstructive HCM, n (%)	170 (21.55)	49 (21.78)	0.941
HFS, n (%)	488(61.85)	199(88.44)	<0.001
P wave duration, ms	111.63 ± 15.75	115.81 ± 21.03	0.013
$PTFV_1$, ms x µV	3490.50 (2101.50, 5562.75)	4770.00 (2256.00, 6732.00)	0.002
PR interval, ms	168.88 ± 29.68	172.87 ± 35.99	0.121
P dispersion, ms	16.59 ± 20.01	24.52 ± 24.55	<0.001
PWA, degree	43.81 ± 21.05	43.12 ± 24.77	0.731
QRS duration, ms	98.94 ± 19.15	102.36 ± 22.12	0.034
QTc interval, ms	457.31 ± 31.25	461.71 ± 33.84	0.090
A-IAB, n (%)	8(1.04)	10(4.44)	<0.001
Frequent PACs, n (%)	38(4.82)	29(12.89)	<0.001
Frequent PVCs, n (%)	37(4.69)	19(8.44)	0.030
Mild MR, n (%)	214 (27.12)	61 (30.67)	0.296
Moderate MR, n (%)	31 (3.93)	13 (5.78)	0.230
Severe MR, n (%)	6 (0.76)	3 (1.33)	0.421
Mild TR, n (%)	40 (5.07)	11 (4.89)	0.913
Moderate TR, n (%)	1 (0.13)	1 (0.44)	0.395

Severe TR, n (%)	1 (0.13)	2 (0.89)	0.126
SAM, n (%)	87(11.03)	27(12.00)	0.683
LVAA, n (%)	7(0.89)	3(1.33)	0.830
CIED implantation, n (%)	56 (7.10)	32 (14.22)	<0.001
Lymphocyte count, x10^9/L	2.06 ± 0.71	1.96 ± 0.74	0.062
Neutrophil count, x10^9/L	4.17 ± 2.15	4.27 ± 2.01	0.537
Platelet count, x10^9/L	212.53 ± 58.99	196.33 ± 54.61	<0.001
NLR	1.89 (1.37, 2.58)	1.91 (1.51, 2.90)	0.035
SII	387.16 (260.12, 560.78)	377.00 (265.57, 568.90)	0.715
ALT, U/L	21.00 (15.00, 32.00)	24.00 (16.00, 32.00)	0.256
AST, U/L	25.32 ± 20.17	29.80 ± 46.74	0.058
TBIL, µmol/L	14.09 ± 7.57	14.91 ± 6.51	0.181
ALB, g/L	42.16 ± 3.60	41.96 ± 3.64	0.509
Creatinine, µmol/L	77.96 ± 88.60	77.21 ± 56.78	0.905
UA, µmol/L	375.95 ± 100.24	370.95 ± 106.37	0.532
TC, mmol/L	4.78 ± 1.07	4.80 ± 1.06	0.887
TG, mmol/L	1.73 ± 1.53	1.52 ± 0.82	0.092
HDL-C, mmol/L	1.18 ± 0.30	1.20 ± 0.28	0.393
LDL-C, mmol/L	2.72 ± 0.77	2.78 ± 0.78	0.351
FPG, mmol/L	6.06 ± 2.30	6.22 ± 2.44	0.374
PT, s	11.14 ± 1.54	11.38 ± 1.75	0.064
APTT, s	25.27 ± 4.96	25.55 ± 4.06	0.468
D-dimer, µg/L FEU	466.20 ± 1287.86	547.01 ± 919.03	0.441
TSH, pmol/L	2.01 (1.28, 2.83)	1.92 (1.29, 3.01)	0.621
FT4, pmol/L	16.22 ± 5.63	16.01 ± 2.69	0.654
FT3, pmol/L	4.78 ± 1.70	4.47 ± 0.70	0.027
CK-MB, µg/L	3.05 ± 3.89	3.46 ± 3.50	0.246
BNP, pg/mL	152.36 (66.72, 276.59)	320.38 (159.97, 598.92)	<0.001
hs-cTnI, ng/L	0.03 (0.01, 0.09)	0.04 (0.02, 0.11)	0.127
HbA1c, %	6.38 ± 1.36	6.56 ± 1.63	0.167
LA-SI, mm	43.00 ± 4.77	45.85 ± 5.11	<0.001
LA-ML, mm	54.52 ± 5.48	58.89 ± 6.35	<0.001
LA-AP, mm	39.42 ± 4.23	42.90 ± 5.35	<0.001
LVEDD, mm	46.31 ± 5.24	46.77 ± 5.15	0.290
LVEF, %	57.76 ± 3.89	56.22 ± 5.81	<0.001
WTmax, mm	18.49 ± 3.80	18.70 ± 3.61	0.476
LVOT-v, m/s	2.51 ± 1.55	2.53 ± 1.57	0.897
E/e'	11.53 ± 5.40	12.99 ± 5.35	0.008
β-blocker, n (%)	754 (95.56)	211 (93.78)	0.270
Non-DHP CCBs, n (%)	332 (42.08)	94 (41.78)	0.936
ARNI/ACEI/ARB, n (%)	229 (29.02)	60 (26.67)	0.490
Diuretics, n (%)	147 (18.63)	56 (24.89)	0.039
SGLT2 inhibitors, n (%)	76 (9.64)	27 (12.00)	0.293
Statins, n (%)	262 (33.21)	55 (24.44)	0.012

Trimetazidine, n (%)	149 (18.88)	36 (16.00)	0.323

HCM, hypertrophic cardiomyopathy; HFS, heart failure symptoms; $PTFV_1$, P wave terminal force in lead V_1; PWA, P wave axis; A-IAB, advanced interatrial block; Frequent PACs, frequent premature atrial contractions; Frequent PVCs, frequent premature ventricular contractions; MR, mitral regurgitation; TR, tricuspid regurgitation; SAM, systolic anterior motion of the mitral valve; LVAA, left ventricular apical aneurysm; CIED, cardiac implantable electronic device; NLR, neutrophil-to-lymphocyte ratio; SII, systemic immune-inflammation index; LA-SI, left atrial superoinferior diameter; LA-ML, left atrial mediolateral diameter; LA-AP, left atrial anteroposterior diameter; LVEDD, left ventricular end-diastolic diameter; LVEF, left ventricular ejection fraction; WTmax, maximal left ventricular wall thickness; LVOT-v, left ventricular outflow tract peak systolic velocity; Non-DHP CCBs, non-dihydropyridine calcium channel blockers; ARNI, angiotensin receptor-neprilysin inhibitor; ACEI, angiotensin-converting enzyme inhibitor; ARB, angiotensin II receptor blocker.

Supplementary Table 2. Full terms of feature labels and abbreviations used in figures.

Feature label/abbreviation	Full term
Age	Age
BNP	B-type natriuretic peptide
CIED	Cardiac implantable electronic device
DDimer	D-dimer
Diuretics	Diuretics
Ee_ratio	E/e’ ratio
frequent_PACs	Frequent premature atrial contractions
frequent_PVCs	Frequent premature ventricular contractions
HCM_family_history	Family history of hypertrophic cardiomyopathy
HFS	Heart failure symptoms
LA_AP	Left atrial anteroposterior diameter
LA_ML	Left atrial mediolateral diameter
LA_SI	Left atrial superoinferior diameter
LVEDD	Left ventricular end-diastolic diameter
LVEF	Left ventricular ejection fraction
Moderate_severe_TR	Moderate-to-severe tricuspid regurgitation
PRInterval	PR interval
P_Dispersion	P wave dispersion
P_Duration	P wave duration
PTFV1	P wave terminal force in lead V_1
PWA	P wave axis
QRSDuration	QRS duration
QTC	Corrected QT interval
Statins	Statins
WT_max	Maximal left ventricular wall thickness

2 Supplementary Figures

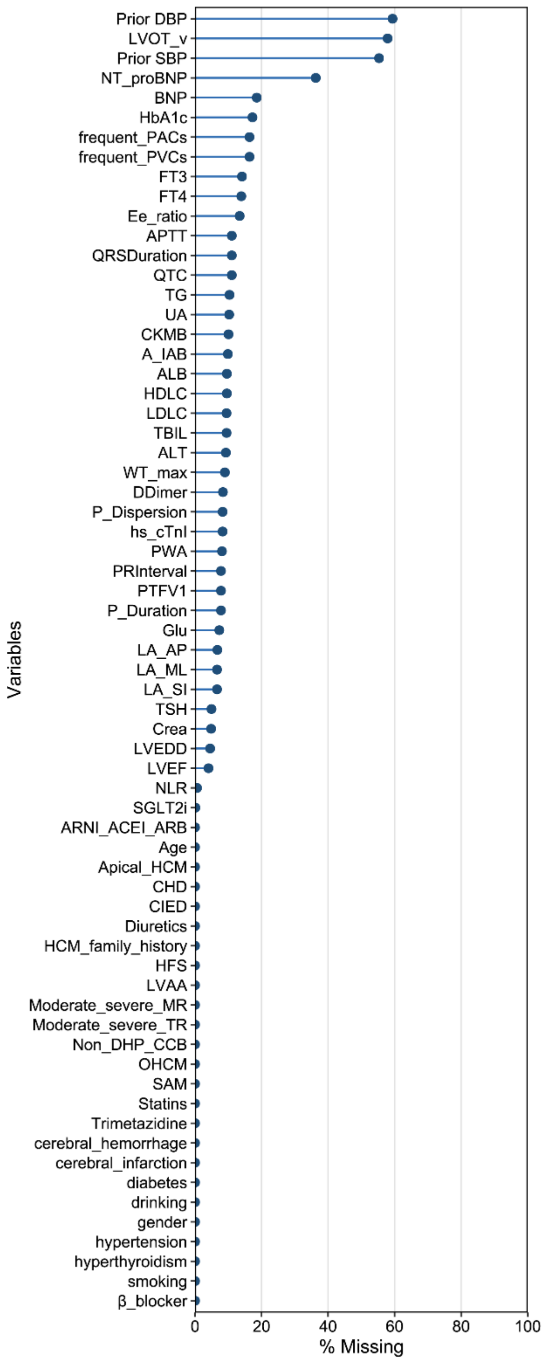

Supplementary Figure 1. Missing percentages of candidate variables before imputation. Full names of feature abbreviations are provided in Supplementary Table 2.

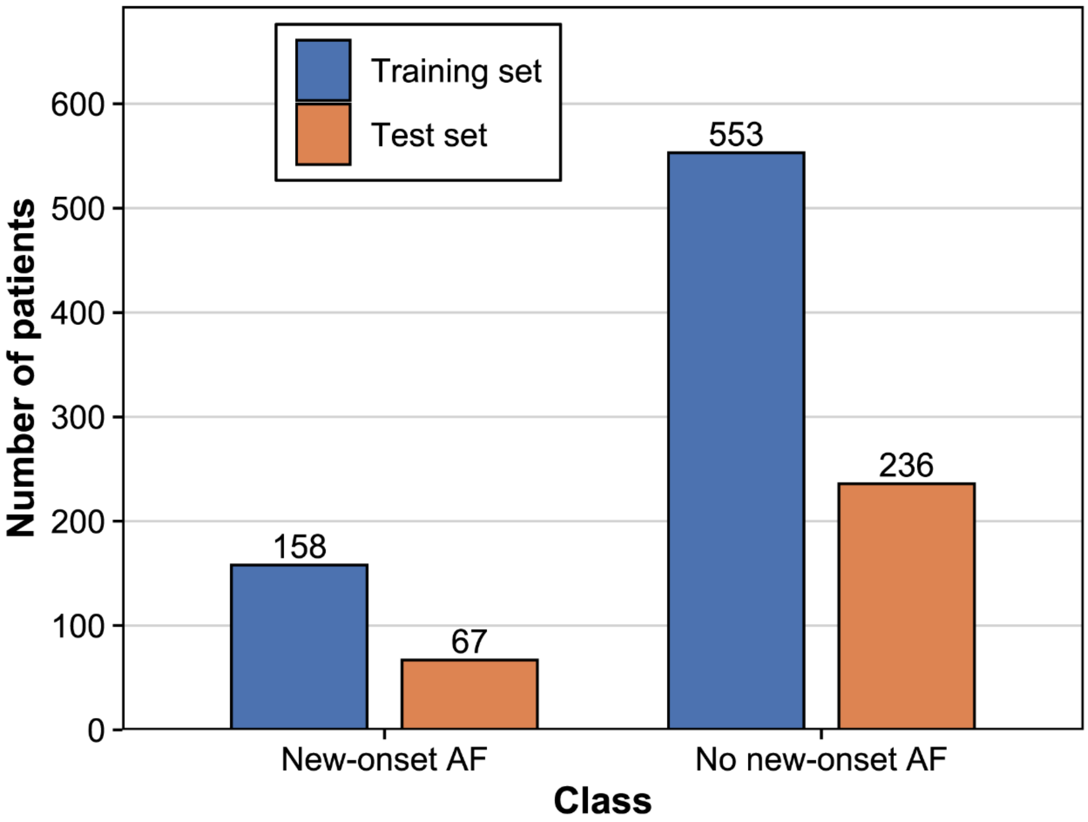

Supplementary Figure 2. Class distribution in the training set and test set. AF, atrial fibrillation.

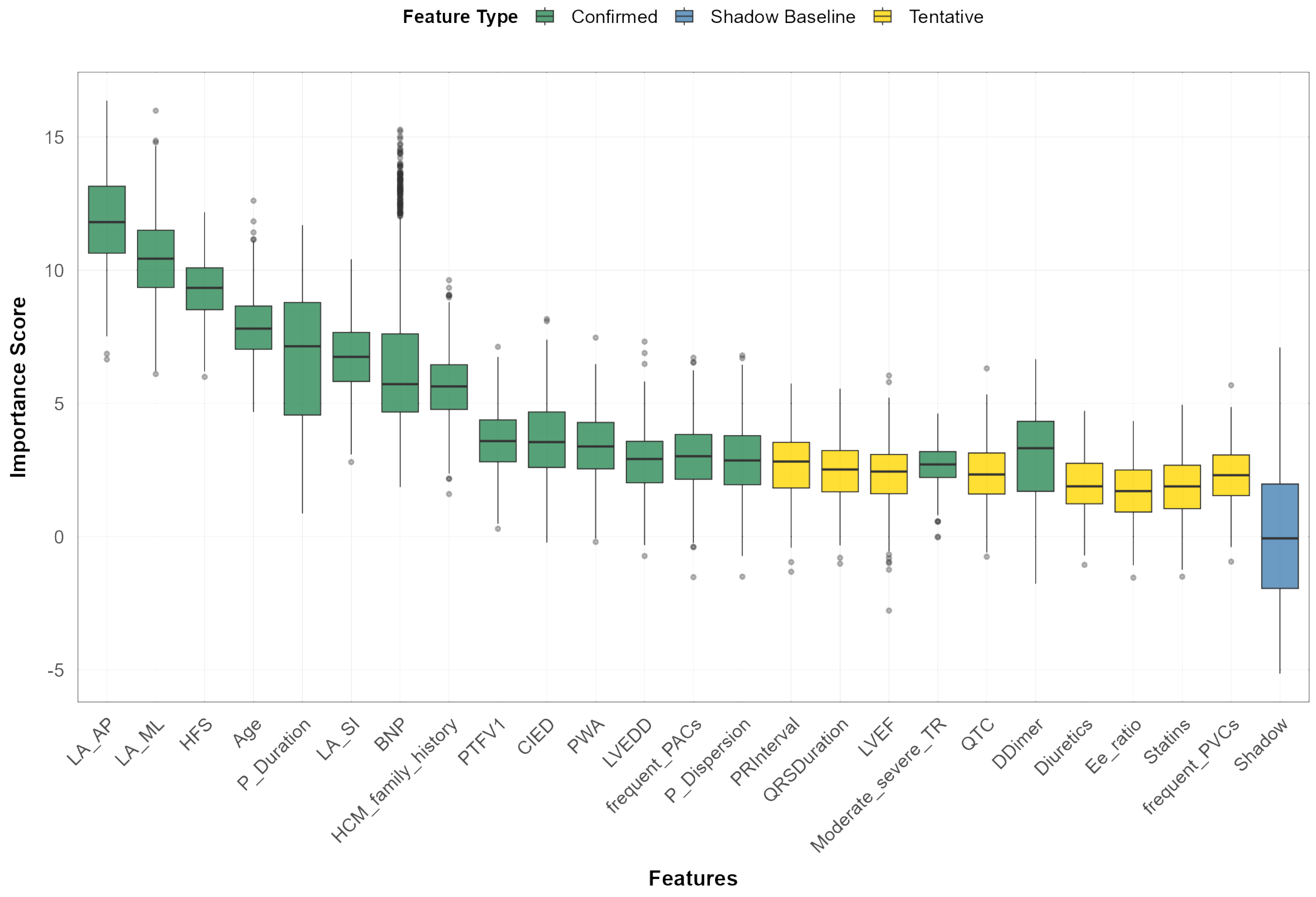

Supplementary Figure 3. Feature importance ranking based on the Boruta algorithm. The Boruta algorithm was applied to identify relevant predictors from all baseline variables. Green boxplots represent confirmed important features, yellow boxplots indicate tentative features requiring further evaluation, and blue boxplots correspond to shadow (randomized) features used as internal references.

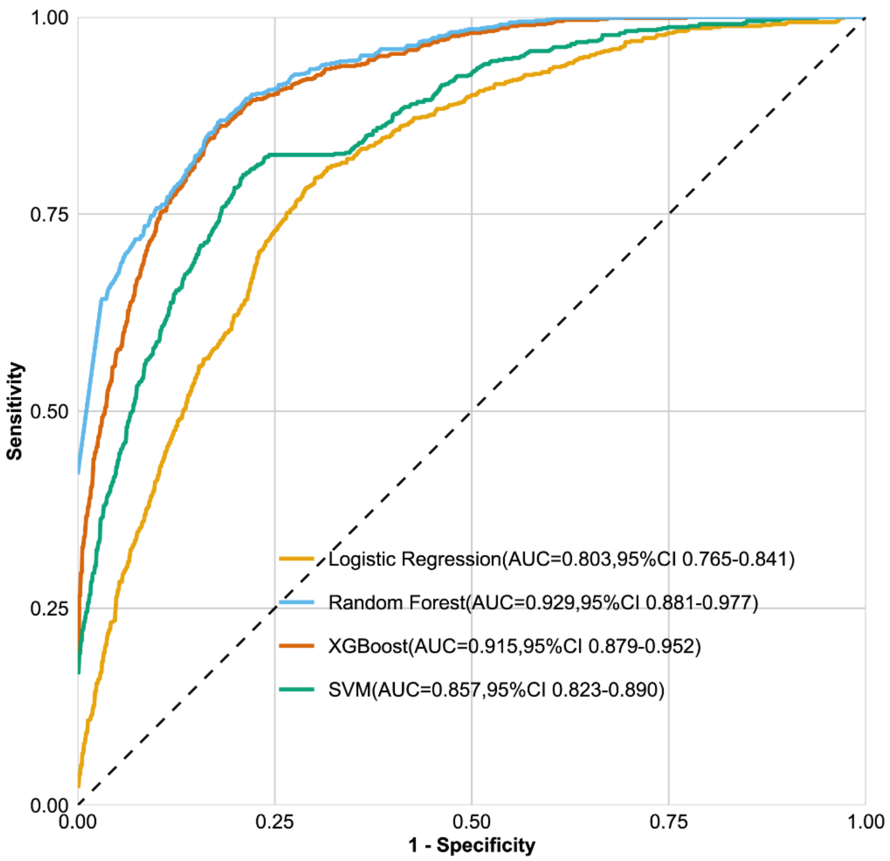

Supplementary Figure 4. ROC curves of four machine learning models based on the training set. XGBoost, extreme gradient boosting; SVM, support vector machine; AUC, area under the curve; CI, confidence interval.

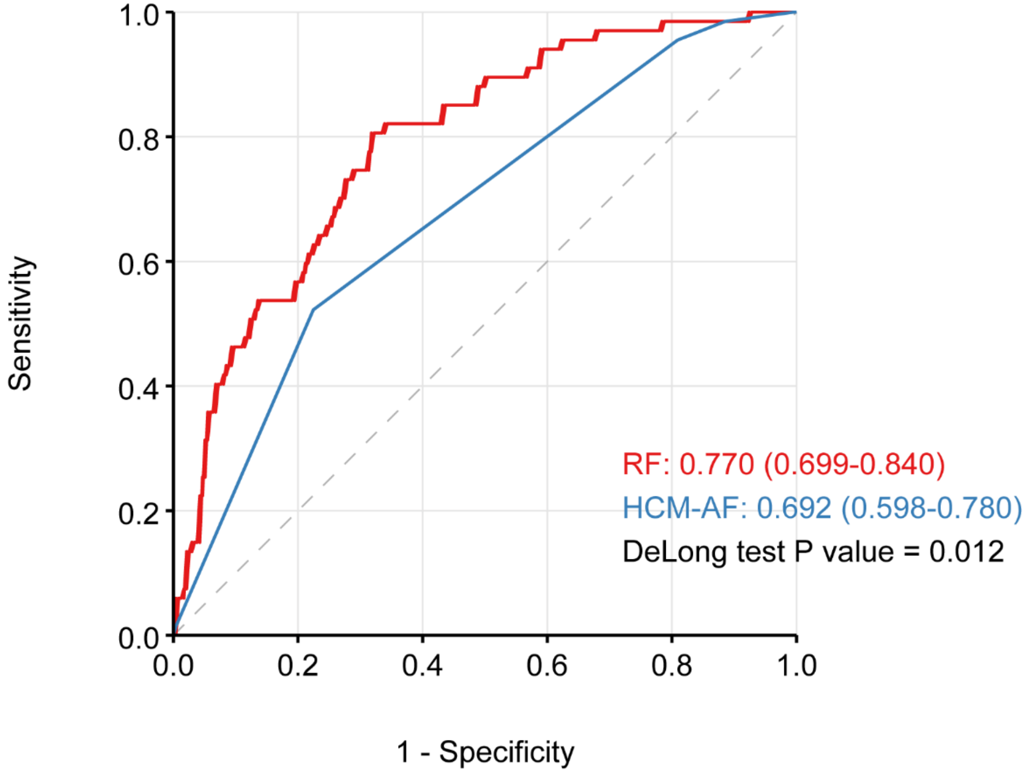

Supplementary Figure 5. ROC curves comparing the Random Forest model and the HCM-AF score for AF prediction.

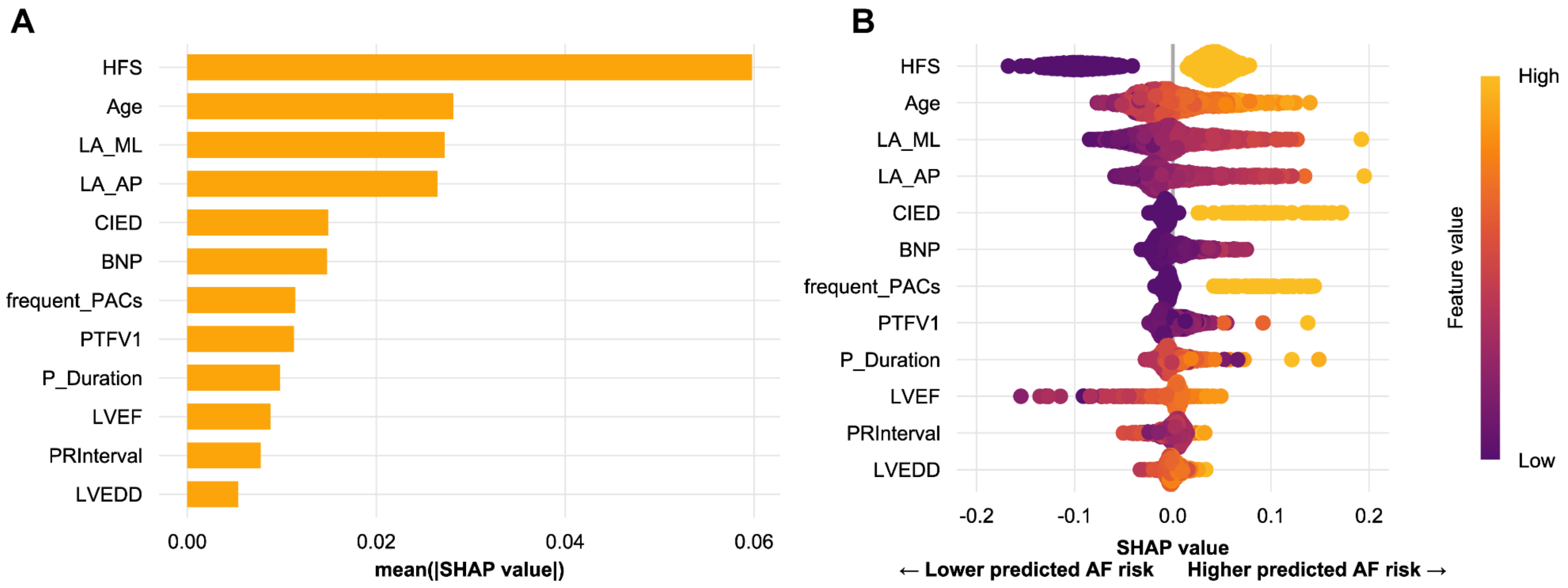

Supplementary Figure 6. SHAP-based interpretation of the Random Forest model in the D1 training set. (A) Mean absolute SHAP value ranking of feature importance. (B) SHAP beeswarm plot showing the contribution and direction of each feature to individual predictions.
